# Genome analysis of the freshwater planktonic *Vulcanococcus limneticus* sp. nov. reveals horizontal transfer of nitrogenase operon and alternative pathways of nitrogen utilization

**DOI:** 10.1186/s12864-018-4648-3

**Published:** 2018-04-16

**Authors:** Andrea Di Cesare, Pedro J. Cabello-Yeves, Nathan A. M. Chrismas, Patricia Sánchez-Baracaldo, Michaela M. Salcher, Cristiana Callieri

**Affiliations:** 1 National Research Council CNR-ISE, Largo Tonolli 50, 28922 Verbania, Italy; 2 Department of Earth, Environmental, and Life Sciences, University of Genoa, 16132 Genoa, Italy; 3 Evolutionary Genomics Group, Departamento de Producción Vegetal y Microbiología, Universidad Miguel Hernández, San Juan de Alicante, Spain; 4 School of Geographical Sciences, University of Bristol, Bristol, BS8 1SS UK; 5 Marine Biological Association of the United Kingdom, The Laboratory, Citadel Hill, Plymouth, UK; 6 Limnological Station, Institute of Plant and Microbial Biology, University of Zurich, Kilchberg, Switzerland

**Keywords:** Picocyanobacteria, *Vulcanococcus limneticus* sp. nov., Nitrogenase genes, Nitrogen fixation, Genomic island, Horizontal gene transfer (HGT)

## Abstract

**Background:**

Many cyanobacteria are capable of fixing atmospheric nitrogen, playing a crucial role in biogeochemical cycling. Little is known about freshwater unicellular cyanobacteria *Synechococcus* spp. at the genomic level, despite being recognised of considerable ecological importance in aquatic ecosystems. So far, it has not been shown whether these unicellular picocyanobacteria have the potential for nitrogen fixation. Here, we present the draft-genome of the new pink-pigmented *Synechococcus*-like strain *Vulcanococcus limneticus.*

sp. nov., isolated from the volcanic Lake Albano (Central Italy).

**Results:**

The novel species *Vulcanococcus limneticus* sp. nov. falls inside the sub-cluster 5.2, close to the estuarine/marine strains in a maximum-likelihood phylogenetic tree generated with 259 marker genes with representatives from marine, brackish, euryhaline and freshwater habitats. *V.limneticus* sp. nov. possesses a complete nitrogenase and *nif* operon. In an experimental setup under nitrogen limiting and non-limiting conditions, growth was observed in both cases. However, the nitrogenase genes (*nifHDK)* were not transcribed, i.e., *V.limneticus* sp. nov. did not fix nitrogen, but instead degraded the phycobilisomes to produce sufficient amounts of ammonia. Moreover, the strain encoded many other pathways to incorporate ammonia, nitrate and sulphate, which are energetically less expensive for the cell than fixing nitrogen. The association of the *nif* operon to a genomic island, the relatively high amount of mobile genetic elements (52 transposases) and the lower observed GC content of *V.limneticus* sp. nov. *nif* operon (60.54%) compared to the average of the strain (68.35%) support the theory that this planktonic strain may have obtained, at some point of its evolution, the *nif* operon by horizontal gene transfer (HGT) from a filamentous or heterocystous cyanobacterium.

**Conclusions:**

In this study, we describe the novel species *Vulcanococcus limneticus* sp. nov., which possesses a complete *nif* operon for nitrogen fixation. The finding that in our experimental conditions *V.limneticus* sp. nov. did not express the *nifHDK* genes led us to reconsider the actual ecological meaning of these accessory genes located in genomic island that have possibly been acquired via HGT.

**Electronic supplementary material:**

The online version of this article (10.1186/s12864-018-4648-3) contains supplementary material, which is available to authorized users.

## Background

The reduction of atmospheric dinitrogen gas (N_2_) to ammonia (NH_3_) is a very energetically expensive process because of the high stability of the dinitrogen molecule, due to the triple bond between the two nitrogen atoms [1]. At the same time nitrogen is a key element for primary productivity in the ocean [2] and nitrogen fixation is a fundamental process in several aquatic environments [3, 4]. A minimum set of six *nif* conserved genes is required in bacteria for nitrogen fixation. Three code for structural and catalytic components (*nifHDK*) and three (*nifENB*) for components involved in the biosynthesis of FeMoco enzymes, which are involved in dinitrogenase activation [5]. Transcription of *nifHDK* can be used as a proxy for nitrogen fixation levels [4].

Diazotrophs are microorganisms capable of converting dinitrogen into ammonia [1]. Cyanobacteria are the only oxygen producing microorganisms able to perform nitrogen fixation [6]. Typically, cyanobacterial nitrogenases are organized in different operons, *nifB-fdxN-nifSU, nifHDK, nifENXW* and *nifVZT* [7]. The nitrogenase complex is irreversibly damaged by oxygen, thus diazotrophic microorganisms have developed different strategies to ensure the success of this process. The filamentous heterocystous cyanobacteria differentiate a specialized nitrogen-fixing cell, the heterocyst, that constitutes an anaerobic site well protected from external oxygen by a thick membrane. Colonial filamentous non-heterocystous cyanobacteria (e.g. *Planktothrix serta*), which lack heterocysts, segregate nitrogen fixation and photosynthesis both spatially and temporally [8, 9]. Non-heterocystous diazotrophic unicellular cyanobacteria (e.g. *Cyanothece, Synechococcus*) evolved a temporal separation strategy, fixing nitrogen during the night and performing photosynthesis during the day, thus separating oxygenic PSII activity from the nitrogenase complex [10].

These last kind of unicellular cyanobacteria have been recognized to be important in marine systems and are potentially abundant enough to significantly contribute to oceanic nitrogen fixation [11, 12]. The presence of a *nif* operon in the cyanobacterial ancestor is controversial: on the one hand it could be acquired by horizontal gene transfer (HGT) after the origin of this clade, probably as consequence of the “fixed nitrogen crisis” between the Archean and Proterozoic era, which led to a lack of available nitrogen in the upper ocean promoting acquisition of *nif* genes from a heterotrophic prokaryote via HGT [13]. Alternatively, a more recent paper [14] proposed that the *nif* genes were present in the cyanobacterial ancestor, repeatedly lost and gained again within the cyanobacterial phylum.

In the light of the central role of nitrogen fixation in biogeochemical cycles, it is crucial to study the *nif* operon in terms of presence, phylogenetic relationships and functionality in strains within the cyanobacterial clades. The ubiquitous unicellular cyanobacteria, *Synechococcus*, is a polyphyletic genus [15] currently being reclassified [16] and is recognized as being of ecological importance in aquatic ecosystems [17]. Yet it has often been neglected regarding nitrogen fixation and only few data are found in the literature on this issue. Only two sequenced *Synechococcus* from Yellowstone hot spring mat [18] were reported to contain the *nif* operon and were described as nitrogen fixing. Certain strains showed nitrogenase activity only under anaerobic conditions [19], while others were active also under microaerobic conditions. This is the case of the *Synechococcus* SF1 isolated from the blades of *Sargassum fluitans* which protects the nitrogenase complex by consuming excess oxygen through Uptake Hydrogenase activity [20, 21]. Another interesting strategy was used by *Synechococcus* RF-1, isolated from a rice field, capable of rhythmic nitrogen-fixing activity [22]. The marine *Synechococcus* strains Miami BG43511 and BG43522 developed a temporal regulation [23]. Nevertheless, there is no evidence for the occurrence of planktonic freshwater *Synechococcus-*like species with the potential for nitrogen fixation, nor for the activity of *nif* genes by RNA transcript quantification.

In order to better understand the possible contribution of freshwater picocyanobacteria to nitrogen fixation we studied *Vulcanococcus limneticus* sp. nov. (formerly *Synechococcus* LL) isolated from a volcanic lake in central Italy, previously sequenced at draft genome level, finding it positive for the *nif* operon. Thus: i) we analyzed the sequences of the genes within the *nif* operon and the phylogenetic relationships between this cluster and others from *Synechococcus* and other cyanobacteria strains, ii) we measured *nifHDK* transcripts under nitrogen limitation conditions and iii) we analyzed the genome to find other ways for this strain to use nitrogen.

## Results and discussion

### *Phenotypic and genomic features of* Vulcanococcus limneticus sp. nov.

In this work we present the genome of *V.limneticus* sp. nov., a novel planktonic strain isolated from the volcanic Lake Albano in central Italy (41°44′47.5″N, 12°40′14.3″E) (Additional file 1: Table S1) [24]. This strain, previously described as *Synechococcus* LL and used in experiments [24, 55], is composed by phycoerythrin-rich cells (Additional file 2: Fig. S2). The genome has a total size of 3,548,882 bp and the GC content of the strain is 68.35%.

### *Phylogenomics of the planktonic* Vulcanococcus limneticus *sp. nov.*

**Fig. 1:**
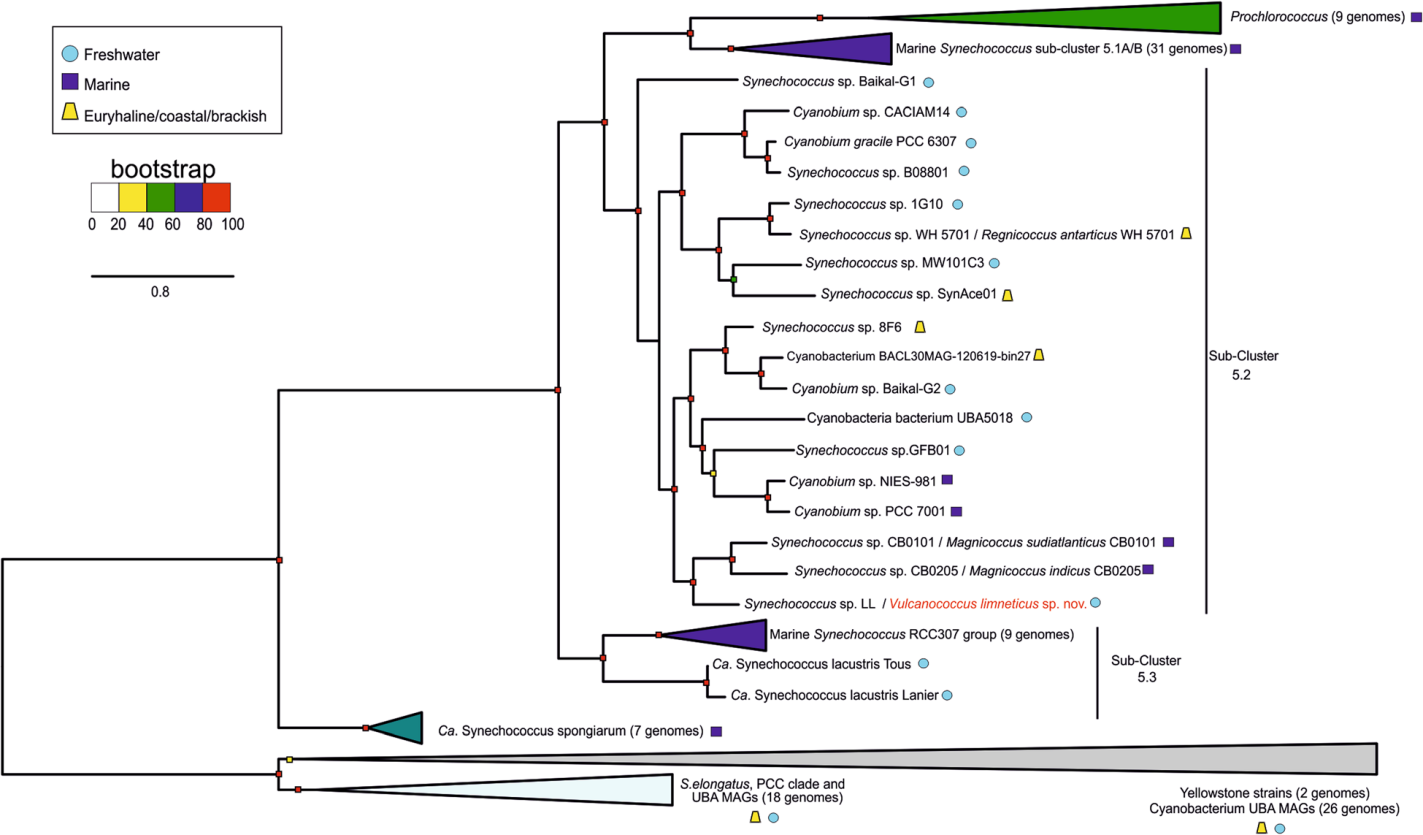
Phylogenomics of *Vulcanococcus limneticus* sp. nov. Two hundred fifty-nine conserved proteins were used to generate a maximum-likelihood phylogenetic tree with *Synechococcus* and *Cyanobium* representatives from marine, brackish, euryhaline and freshwater habitats together with the novel *V.limneticus* sp. nov. *Prochlorococcus* genomes were also added into the phylogeny

Several studies in the past few years reported that *Synechococcus* and *Cyanobium* sub-clusters 5.2 and 5.3 comprise marine, brackish, euryhaline and freshwater strains [15, 25, 26], whilst sub-cluster 5.1 solely contains marine representatives [27–29]. Additionally, reports based on 16S rRNA genes of freshwater strains have revealed the presence of 13 non-marine clusters inside the 5.3 and 5.2 sub-clusters [24, 30]. Other phylogenomic studies have showed that *Synechococcus* is not a monophyletic group [15], and only recently new classifications and novel genera within the traditionally considered *Synechococcus* clade have been proposed [16]. Based on our phylogenomic results evaluated with PhyloPlAn tool [31] with a total of 259 universal markers, we noted that the planktonic *Vulcanococcus limneticus* sp. nov. falls inside the sub-cluster 5.2 close to the estuarine/marine strains *Synechococcus* CB0205 and CB0101 (isolated from Chesapeake Bay) (Fig. 1). Recent phylogenomic studies led to genus proposal of *Magnicoccus* for CB0205 and CB0101 strains [16], and it appears that our strain affiliates close to these representatives from this new genus. Nonetheless, it presents less than 79% of average nucleotide identity (ANI) and less than 72% of average amino acid identity (AAI) with closest strains as GFB01 and CB0201, hence is the first representative of a novel picocyanobacterial genus. Genome-to-genome DNA hybridization (GGDH), ANI and AAI values with closest species are shown in Additional file 3: Table S2. The enormous diversity seen in sub-cluster 5.2 opens new perspectives for picocyanobacterial evolution, once that studies on a novel pigment gene in the Baltic Sea raised the possibility that picocyanobacteria of the subcluster 5.2 originated in freshwater sources ([32]; P.Sánchez-Baracaldo et al. unpublished).

### *Nitrogenase operon in the freshwater strain* Vulcanococcus limneticus sp. nov

**Fig. 2:**
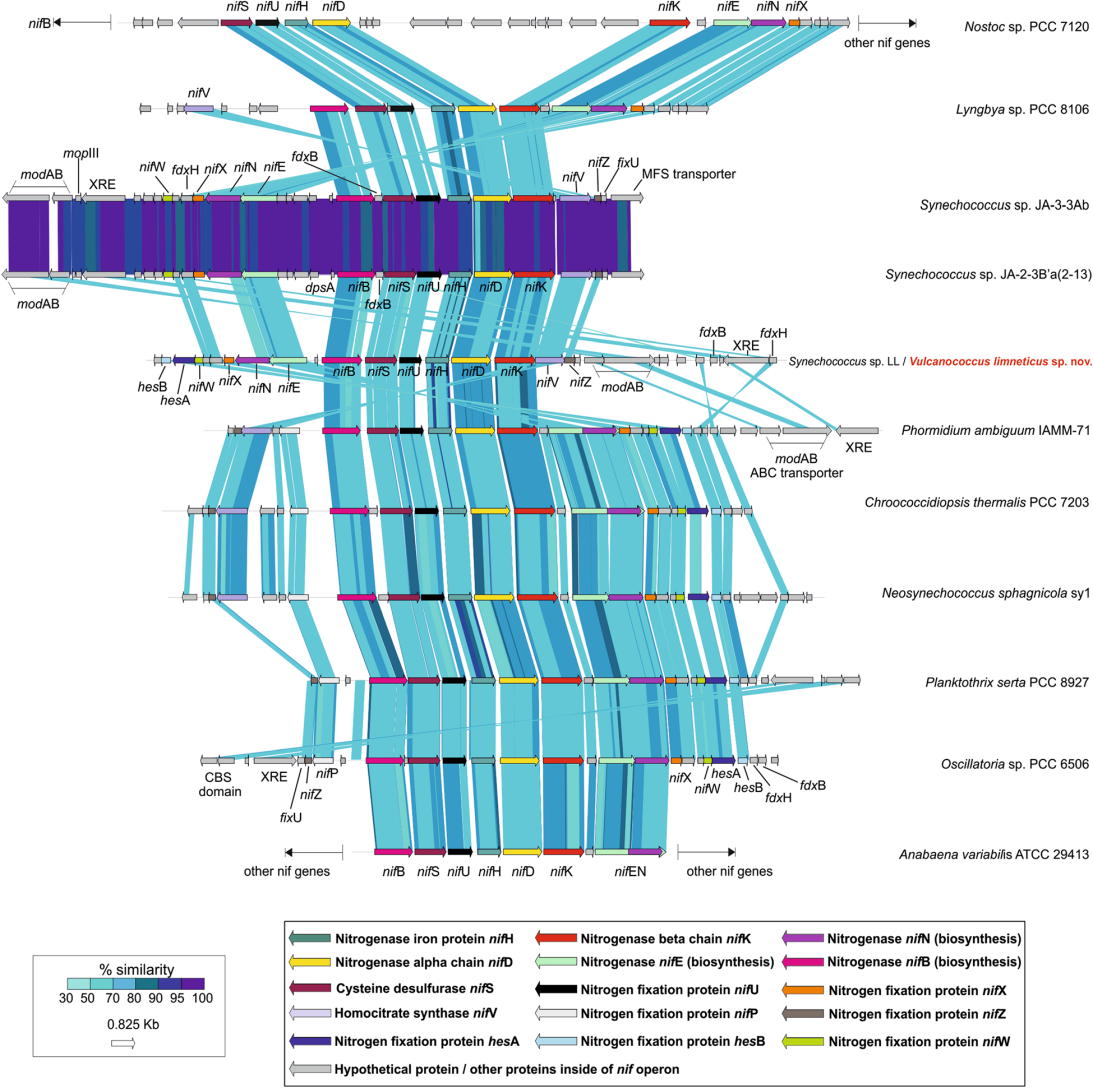
Structure and similarity of the nitrogenase operon among different cyanobacteria. Comparison made with TBLASTX with > 50% similarity hits and 50 bp alignment lengths. The different nitrogenase proteins from *nif* operon are colour coded. Hypothetical and other auxiliary genes present inside the operon *nif* or elsewhere on the genome of the compared strains (such as ferredoxins *fdx*HB, XRE transcriptional regulator, *fix*U protein or molybdenum ABC-transporter) are coloured in grey. In the cases of the heterocystous strains *Nostoc* sp. PCC 7120 and *Anabaena variabilis* ATCC 29413 only a minimum set of *nif* genes is shown, being the rest of the *nif* genes located elsewhere in their genomes

**Fig. 3:**
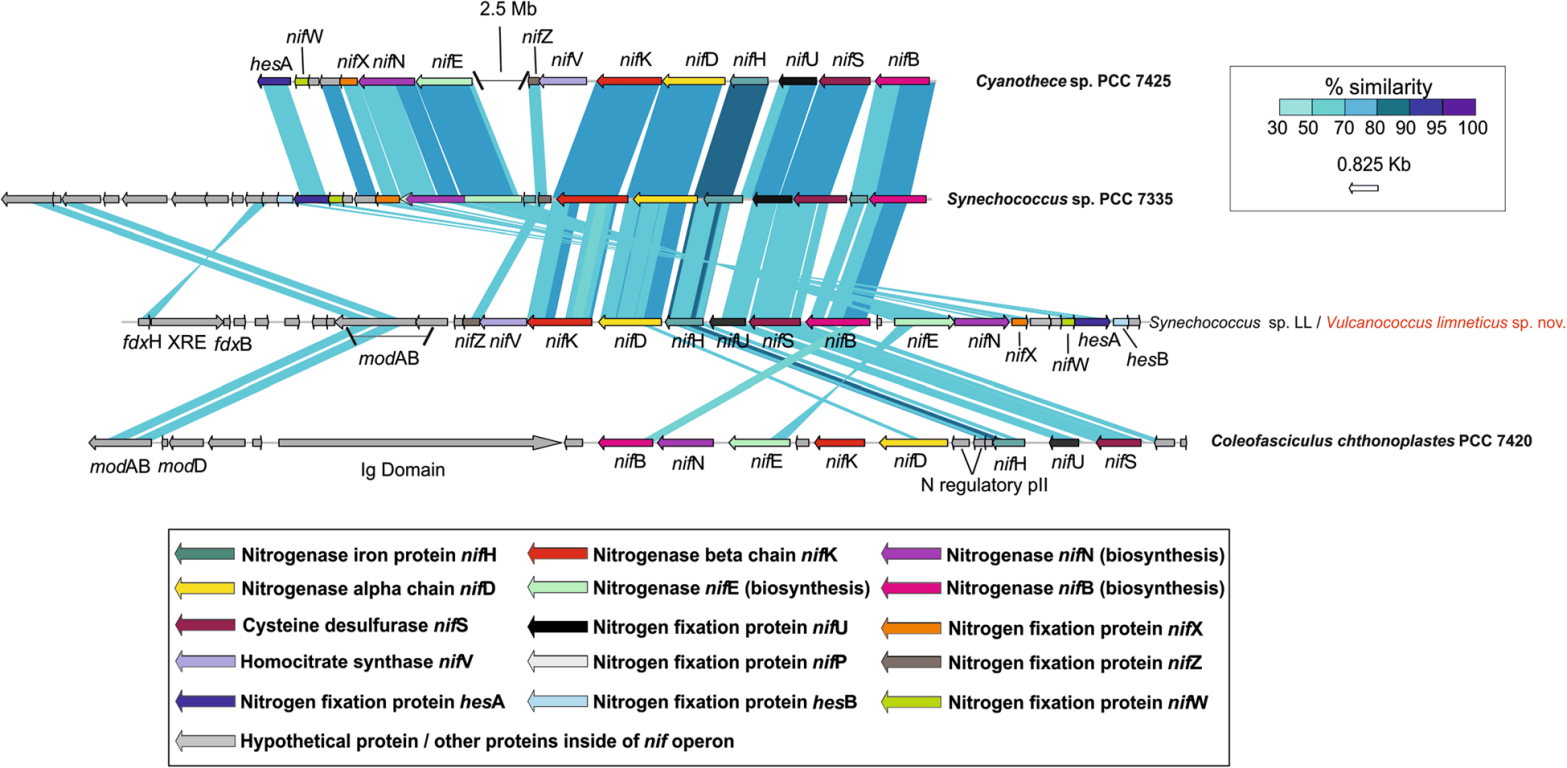
Structure and similarity of the nitrogenase operon among different anaerobic cyanobacteria and *Vulcanococcus limneticus* sp. nov. Comparison made with TBLASTX with > 50% similarity hits and 50 bp alignment lengths. The different nitrogenase proteins from *nif* operon are colour coded. Hypothetical and other auxiliary genes present inside the operon *nif* or elsewhere on the genome of the compared strains (such as ferredoxins *fdxHB*, XRE transcriptional regulator, *fixU* protein or molybdenum ABC-transporter) are coloured in grey. In the case of *Cyanothece* sp. PCC 7425 we show the similarity of different *nif* gene clusters separated by 2.5 Mb in the genome. A minimum set of *nif* genes is shown in the anaerobic cyanobacteria compared to *V.limneticus* sp. nov., being the rest of the *nif* genes located elsewhere in their genomes

**Fig. 4:**
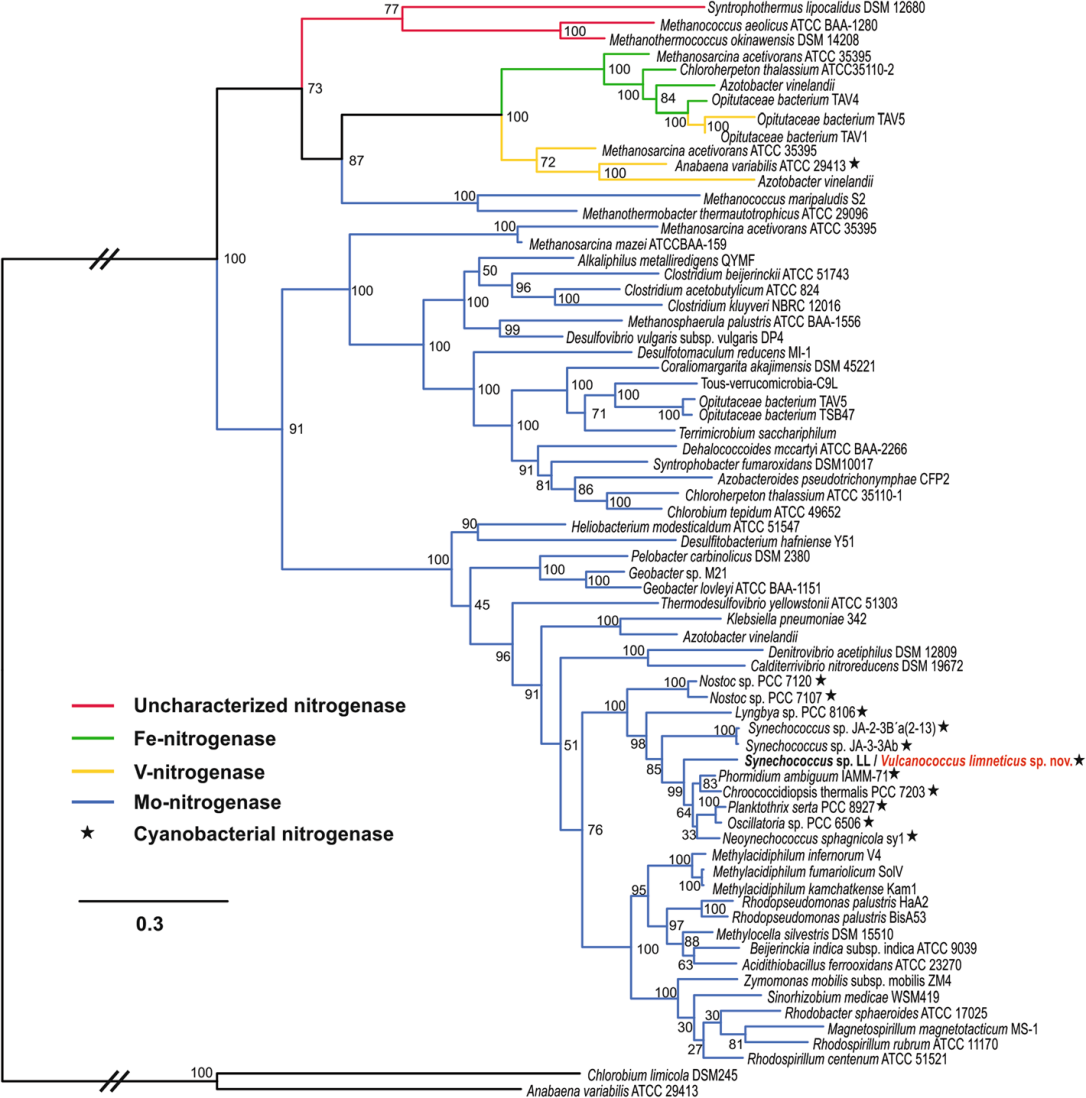
Phylogeny of the NifHDK protein concatamer. Different molybdenum, vanadium and iron nitrogenases from different bacteria are represented. Concatenations of paralogous proteins involved in the synthesis of chlorophyll/bacteriochlorophyll (Bch/ChlLNB) were used to root the phylogeny. The novel nitrogenase from the freshwater *Vulcanococcus limneticus* sp. nov. is red coloured. Cyanobacterial NifHDK are represented with a star symbol

Here, for the first time, we show the presence of nitrogenase operon in the new planktonic freshwater picocyanobacteria *V.limneticus* sp. nov. As shown in Fig. 2, *V.limneticus* sp. nov. exhibits a complete *nif* operon with *nifHDK* catalytic subunits, *nifBSU*, *nifENXW* biosynthetic proteins and the additional subunits *hesB, hesA, nifV, nifZ, fixU*, the transcriptional regulator of *nif* operon XRE (xenobiotic response element), the molybdenum transporter *modAB* and the ferredoxins *fdxB* and *fdxH*. The latter was described as important for the maximum nitrogenase activity in heterocystous cyanobacteria as its inactivation delays nitrogen fixation [33], but it is not essential for their growth [34]. Only *nifP* was absent from this strain. We compared the structure of the *nif* operon with some representatives of cyanobacteria and provided a phylogeny of the *nifHDK* concatamer among different molybdenum, vanadium and iron nitrogenases from different bacterial phyla representatives as previously described [35–38]. Compared to the rest of cyanobacteria (Fig. 2 and Fig. 3) the heterocystous *Anabaena variabilis* and *Nostoc* display a completely different organization of the *nif* operon with some subunits located elsewhere in their genomes. *V.limneticus* sp. nov. contains a slightly different *nif* structure compared with the rest of the other strains, although the closest gene organization is observed in the Yellowstone Hot Springs *Synechococcus* JA-3-3Ab and JA-2-3Ba [18], where the structure of *nifBSUHDKVZ* is maintained except for the insertion of the ferredoxin *fdxB* in these strains between *nifB* and *nifS* subunits. The gene organization compared to anaerobic cyanobacteria also displays different arrangements (Fig. 3), especially compared to the unicellular *Cyanothece* sp. PCC 7425 [39]. The novel. V*.limneticus* sp. nov. nitrogenase affiliates with a cluster of molybdenum cyanobacterial nitrogenases (Fig. 4), although it appears quite distant from heterocystous strains like *Anabaena* and *Nostoc* and forms a different branch from *Synechococcus* JA-3-3Ab and JA-2-3Ba, and other strains like *Planktothrix serta* PCC8927, *Phormidium ambiguum* IAMM-71 and *Chroococcidiopsis thermalis* PCC 7203, *Oscillatoria* sp. PCC 6506, and *Neosynechococcus sphagnicola* sy1. In general, except for the high similarity (90–100%) of the *nif* operon for *Synechococcus* JA-3-3Ab and JA-2-3Ba, we observed low levels of genetic identity (from 50 to 80%) despite the well conserved synteny of the different subunits between the different cyanobacteria tested, confirming an enormous genetic diversity of nitrogenases in the cyanobacterial phylum.

### *Evidence of HGT of the* nif *operon in* V.Limneticus sp. nov.

A previous study carried out in the cyanobacterium *Microcoleus chthonoplastes* showed acquisition of its *nif* operon by HGT [40]. We found some genomic features inside the genome of this planktonic strain that could provide evidence for a HGT of its *nif* operon. Following metagenomic fragment recruitment methods described in other publications for freshwater *Synechococcus* [26] we noted the highest abundance of *V.limneticus* sp. nov. in two Amazon lakes from which metagenomic studies were carried out [41]. This strain was not detected significantly (above species level, > 95% of ANI) in other available freshwater metagenomes from all over the world. It must be noted the lack of metagenomes of volcanic lakes. As depicted from Additional file 4: Fig. S1, we observed that *V.limneticus* sp. nov. genome is fully covered both above the species level (95%) and between 80 and 95% of identity values, which confirms that *V.limneticus* sp. nov. clones from the same species (> 95%) are present in these tropical Amazon lakes together with closely and distantly related species (80–95%). The regions of low coverage in these metagenomes, also called genomic islands, were associated to LPS biosynthesis, genetic mobile elements or other phage defense systems, as previously described in marine *Synechococcus* [29]*.* However, we noted that *nif* operon was also associated with a genomic island, being absent from the majority of the *Synechococcus* population and apparently rare in the tropical Lake Ananá and Mancapuru Great lake. The GC content of the contig containing the *nif* operon (26,472 bp and 60.54%) also contrasts with the GC content of the strain (68.35%). The genes flanking the *nif* operon contain an average GC content of 65–67%, whilst the majority of *nif* genes present lower values from 56 to 61% and give top BLAST hits to heterocystous, filamentous and other Cyanobacteria as *Leptolyngbya, Lyngbya, Phormidium, Oscillatoria, Microcoleus, Kamptonema, Calothrix, Pseudoanabaena* or *Tolypothrix* species. Moreover, we noted a relatively high amount of transposases and mobile elements in *V.limneticus* sp. nov. (52), mostly at the beginning and at the end of the different contigs, which confirms that this strain is genetically prepared for gene transfer events. These features may reinforce the theory that this planktonic strain may have obtained, at some point of its evolution, the *nif* operon by HGT from a filamentous or heterocystous cyanobacterium.

### *Expression of* nifHDK *genes*

To estimate the expression level of the *nifHDK* genes of *V.limneticus* sp. nov.we measured RNA transcripts using real time PCR in two experimental conditions: in the presence and absence of nitrogen. *V.limneticus* sp. nov.was able to grow in both conditions (Additional file 2: Fig. S2). However, the RNA transcript quantification of the *nifHDK* genes gave negative results or comparable to those of the non-reverse transcribed RNA (NORT) (Additional file 5: Table S3). Considering these results, we can assume that *nifHDK* genes were not expressed in our experimental conditions. We must also consider that the 16S rRNA gene was always expressed with threshold cycle values between 18.7–19.4 for all the replicates with the CT values for the NORT negative or between 31.7–34.8 in presence and absence of nitrogen (Additional file 5: Table S3). Thus, although *V.limneticus* sp. nov. is able to grow in nitrogen limiting conditions, we might speculate that this strain is not able to perform nitrogen fixation, at least in our experimental conditions (not excluding the possibility of the nitrogen fixation capability by *V.limneticus* sp. nov. in anaerobic conditions). We acknowledge the need to test the transcript quantification of the *nifHDK* genes in other laboratory conditions (e.g. in anaerobic conditions, during other hours of the diel cycle) to make our results more comprehensive. We recognized some similarities with the filamentous, non-heterocystous cyanobacterium *Microcoleus chthonoplastes* that possesses *nif* genes but is unable to express nitrogenase in culture [40].

### *Possible alternative nitrogen sources for* V.Limneticus sp. nov. *under nitrogen limiting conditions*

**Fig. 5:**
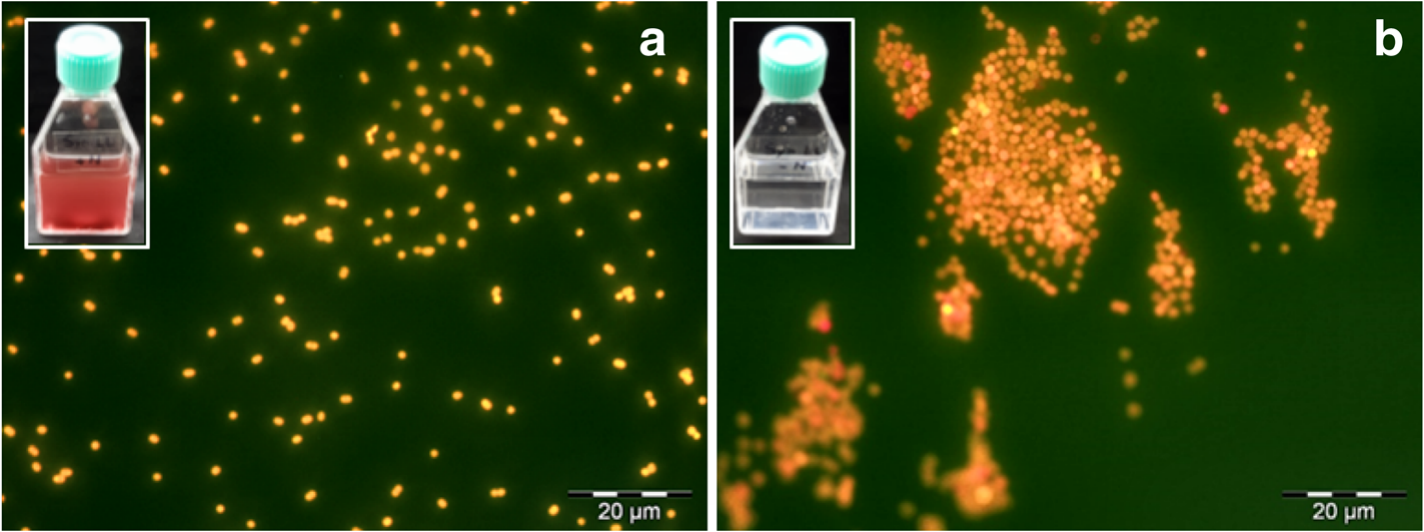
Microphotography at the epifluorescence microscopy (Zeiss Axioplan, 787.5×, blue excitation) of *Vulcanococcus limneticus* sp. nov. after one month of cultivation in a media with nitrogen (**a**) and without nitrogen (**b**). The cultures in the flasks are shown in the upper corner.

When *V.limneticus* sp. nov. is under nutrient limitation, it accumulates glycogen (observed in our –N culture, Additional file 6: Fig. S3) and the phycobilisomes are specifically and rapidly degraded, in a process known as bleaching or chlorosis [42]. We noticed that when keeping the cultures for a longer time-span (one month), the flasks of the treatment without nitrogen bleached while the treatment with nitrogen were of an intense pink colour (Fig. 5). The cells in the –N treatment showed a strong aggregation and a lack of yellow fluorescence under blue excitation (Fig. 5). This phenomenon suggests a loss of the phycobilin pigments, likely indicating a use of the pigments as nitrogen reserve. The degradation of phycobilisomes under nitrogen starvation requires the activity of alanine dehydrogenase (*ald* gene) producing sufficient amounts of ammonia [43]. This gene was found in the *V.limneticus* sp. nov. genome pointing to other mechanisms of nitrogen acquisition. The *V.limneticus* sp. nov. genome harbored multiple mechanisms to incorporate ammonia into the cell. For instance, we detected the ubiquitous and widespread ammonia assimilatory pathway via glutamine synthetase, ferredoxin-dependent glutamate synthetase, glutamine amidotransferase, nitrogen regulatory protein pII and amt transporters. Cyanate hydrolysis has been reported in marine *Synechococcus* and *Prochlorococcus* under nitrogen depleted conditions [44]. Interestingly, we detected the cyanate hydratase (CynS) that catalyses the transformation of cyanate into ammonia and CO_2_, which could provide an evolutionary advantage of *V.limneticus* sp. nov. under nitrogen limitation in Lake Albano. We also observed the nitrate and nitrite ammonification pathway as an alternative source to get ammonia; we detected a nitrate assimilatory reductase (which catalyses the transformation of nitrate to nitrite) and an ammonia ferredoxin oxidoreductase or nitrite reductase (which catalyses the transformation of nitrite into ammonia). Together with these enzymes, we also found two nitrate ABC transporters and four nitrate/sulfonate ABC transporters that could also be used for the uptake of sulfonates and alkanesulfonates.

### *Other metabolic features of* V.Limneticus sp. nov.

It was reported that in nitrogen-fixing cyanobacteria hydrogen is synthesized as a by-product of nitrogenase activity and is further oxidized by a hydrogenase [7, 8]. There are two types of hydrogenases in cyanobacteria: an uptake hydrogenase (HupSL) which catalyses H_2_ consumption and is present in almost all nitrogen-fixing strains [45] and a bidirectional hydrogenase (HoxFUYH) which is involved in both hydrogen synthesis and oxidation but seems to be unrelated to the nitrogen-fixing process [46]. In *Synechocystis* PCC 6803, the bidirectional HoxEFUYH hydrogenase functions for H_2_ uptake or production [47]. In *V.limneticus* sp. nov. we detected only this bidirectional hydrogenase (HoxEFUYH). This is surprising since almost all nitrogen-fixing cyanobacteria possess also the uptake hydrogenase (HupSL) except for *Synechococcus* BG 043511 [45]. Hence, it appears that in some strains of N-fixing *Synechococcus* there is an absence of the uptake hydrogenase. The recycling of hydrogen by these type of enzymes generates an anoxic environment necessary for nitrogenase activity [48] and the H_2_ production could be assumed both by nitrogenases and bidirectional hydrogenases [46].

Regarding sulfur metabolism, we observed mechanisms for uptake and utilization of alkanesulfonates in *V.limneticus* sp. nov. Two alkanesulfonate monooxygenases and six sulfonate ABC transporters (TauABCD and ssuEADCB) were detected in the genome. These organosulfur compounds were also reported to be degraded by freshwater Actinobacteria [49] and *Achromobacter* or *Rhodococcus* strains [50]. The utilization of these sulfur sources by our picocyanobacterium is ecologically relevant since sulfonates tend to accumulate in soils, rivers, groundwater [51] and marine sediments [52] and some of them like atmospheric methanesulfonates [53] or herbicides [51] are considered contaminants and hazardous compounds for the environment and very harmful for animals and humans. We also found two different clusters of the specific freshwater sulfate Cys transporter which was previously reported in freshwater *Cyanobium* and *Synechococcus* [26]. A chitinolytic enzyme and key enzymes for the N-acetylglucosamine utilization (NAG core enzymes *nagA*, *nagB* and the NAG kinase *nagK*) were also detected in the genome, which suggests that *V.limneticus* sp. nov. may also be capable of chitin degradation. All these features enhance the ecological relevance of the novel *V.limneticus* sp. nov.

## Conclusions

This study shows that *Vulcanococcus limneticus* sp. nov. possess a complete nitrogenase and *nif* operon, possibly acquired by HGT and located in a genomic island. However, in our experimental conditions, *V.limneticus* sp. nov. did not express the *nifHDK* genes. We cannot eliminate the possibility that in other conditions the *nifHDK* genes might still be expressed. Nevertheless, the presence of genes coding for different enzymes in the *V.limneticus* sp. nov. genome like alanine dehydrogenase or hydrogenase points to other pathways to incorporate ammonia, like direct ammonia assimilation from phycobilisome degradation, nitrate/nitrite ammonification or cyanate hydrolysis, which are energetically less expensive for the cell.

### Description of *Vulcanococcus limneticus* sp. nov

*Vulcanococcus* (Vul.ca.no.coc’cus*,* L. masc. n. *vulcanus* volcan [referring to the volcanic region from which the organism was isolated]; N.L. masc. n*. coccus* of spherical shape); *limneticus* limnetic (lim.ne.ti’cus, L. masc. Adj. [referring to lacustrine origin of the organism]).

The isolation source was the freshwater volcanic Lake Albano located in central Italy. It is composed by aerobic gram-negative non-motile cells approximately 0.97 ± 0.21 μm long and 0.76 ± 0.12 μm wide (volume: 0.36 ± 0.19 μm^3^). It can form microcolonies of 10–20 cells. *Vulcanococcus limneticu*s sp. nov. presents a G + C content of 68.35 mol%. The genome has a total size of 3,548,882 bp. The strain grows optimally in BG11 media for freshwater cyanobacteria at neutral pH and between 19 and 25 °C at low light (10–15 μmol photons m^− 2^ s^− 1^). The strain has a maximum absorbance at 573 nm typical of phycoerythrin and develops a type IIB intense pink-red pigmentation.

## Methods

### Strain characteristics

The strain used for this experiment was a phycoerythrin-rich (PE) picocyanobacteria [24]. This picocyanobacterium has been isolated from a volcanic lake in central Italy (Lake Albano, Additional file 1: Table S1) using cycloheximide (with a final concentration of 3 mmol l^− 1^) to eliminate the picoeukaryotes. Then the culture was purified by sorting (InFlux V-GS flow cytometer, Becton Dickinson Inc.) one single cell each into wells of 96-well plates containing 100 μl of BG11 substrate and kept in a thermostat at 18–20 °C at low light (10–15 μmol photons m^− 2^ s^− 1^) [24]. In this way we obtained a monoclonal culture (derived from one single mother cell) not axenic (with associated microbiome, [54]). This strain has been used in different experiments [24, 55] and was named *Synechococcus* LL based on 16S rDNA. The cells with phycoerythrin are well recognized by the flow cytometer, forming a defined cloud in the cytograms with signal collected in orange and red channels [55]. Recently it has been sequenced for the entire genome (accession number NQLA01000000, Sanchez-Baracaldo et al.*,* submitted) and following the recent *Cyanobacteria* classification scheme [16] based on GGDH, ANI and AAI delineation parameters (Additional file 3: Table S2) it belongs to a new species from a novel genus and it has been renamed as *Vulcanococcus limneticus* sp. nov.

### Genome assembly

DNA was extracted using AXG (Machery-Nagel, Düren, Germany) gravity flow columns as per the manufacturer’s protocol. Library prep was performed with the Illumina TruSeq Nano DNA Library Preparation Kit (Illumina, San Diego, CA) and sequenced on an Illumina Hi-Seq 2500. Reads were trimmed using Trimmomatic v0.32 [56] before being assembled using SPAdes v3.5.0 [57]. Contaminating sequences were removed by identifying cyanobacterial contigs with a database of core cyanobacterial genes [58] using tBLASTn v2.2.30+ (e-value threshold of 1e^− 10^) and visualising using Bandage v0.07 [59] as previously described [60]. The final assembly consisted of 160 contigs and is deposited on NCBI GenBank under the accession number NQLA01000000.

### Phylogenomic trees

A reference protein-concatenate-based tree with a total of 259 universal markers based on PhyloPlAn tool [31] was created, using a total of 111 *Synechococcus* and *Cyanobium* genomes originating from multiple habitats. Nine *Prochlorococcus marinus* were also used to complete the phylogeny.

### Single gene trees

A NifHDK protein concatamer tree was constructed with NifHDK concatamers from a wide range of bacteria as previously described [35–38]. Sequences were aligned using MAFFT [61] and trees were constructed with FastTree2, using JTT + CAT model, a gamma approximation and 100 bootstraps.

### *Comparison of the nitrogenase* nif *operon among different Cyanobacteria*

The structure and similarity of the nitrogenase subunits for the different compared cyanobacteria, including the novel planktonic *Vulcanococcus limneticus* sp. nov., was assessed by tBLASTX [62] with > 50% similarity hits and 50 bp of alignment lengths.

### Genome annotation and metabolic pathways

We performed BLAST [62], BATCH web CD-Search Tool [63] and RAST annotation server tools [64] in order to detect different genes and metabolic pathways in *V.limneticus* sp. nov.

### *Metagenomic fragment recruitment of Vulcanococcus limneticus* sp. nov. *on Amazon lake datasets*

We performed recruitment plots following methods from other publications [26]. We used Lake Ananá and Mancapuru Great lake metagenomic datasets to evaluate the presence of *V.limneticus* sp. nov. and its low covered genomic islands in the tested metagenomes [41].

### Experimental set-up

We carried out a laboratory experiment with *Vulcanococcus limneticus* sp. nov., in conditions in which it grows very well, by simply using two treatments, with (+N) and without (-N) nitrogen in the culture media, to measure *nifHDK* transcripts under nitrogen limitation conditions. In this way, we were able to use a non-axenic culture to recognize the activation of the *nifHDK* gene for the nitrogenase activity specific for *V.limneticus* sp. nov. The culture media was specific for cyanobacteria with nitrogen present as NaNO_3_ at a final concentration of 8 mmol L^− 1^ of N in the medium and N: P of 20 [65]. We added 1 ml of a dense culture (inoculum) to 50 ml of each medium with (+N) and without (-N) nitrogen to reach starting abundances of *V.limneticus* sp. nov. around 8 × 10^5^ cells ml^− 1^. The volume of the inoculum was low, so that the nitrogen added in the –N treatment was considered negligible. Each treatment was performed in triplicate, in semi-continuous cultures, kept in the same condition of maintenance in a culture room at 20 °C, with a 12 h light: 12 h dark diel-cycle using cool white fluorescent tubes at an intensity of 20 μmol photons m^− 2^ s^− 1^. Each day at the end of the light cycle and at the beginning of the dark cycle (Additional file 4: Fig. S1) 0.5 ml were taken from each treatment and fixed for counting (0.2 μm filtered formaldehyde at 1% final concentration). We followed the growth of the culture calculating the daily growth rate and when the culture was in exponential phase (on ninth day), we sampled for the RNA analysis at the beginning of the dark cycle as previously suggested [66]. The culture flasks were incubated at the same experimental conditions for one month to control the state of the culture on a longer time-span.

Counting was performed on a flow cytometer Accuri C6 (Becton Dickinson Inc., New Jersey, US), equipped with a 50 mW laser emitting at a fixed excitation wavelength of 488 nm. The instrument provides light scattering signals (forward and side light scatter named FSC and SSC, respectively), green fluorescence (FL1 channel = 533/30 nm), orange fluorescence (FL2 channel = 585/40 nm) and red fluorescence (FL3 channel > 670 nm and FL4 channel 675/25). For counting we used FL2-H vs FL3-H which better singled out the phycoerythrin-rich *V.limneticus* sp. nov. and allowed optimal gating design.

White polycarbonate filters (Poretics, 0.2 μm pore size) were used for microscopic evaluation of the cells at the beginning of the experiment, at the sampling for RNA analyses and at the end of the experiment. We used a Zeiss Axioplan microscope equipped with an HBO 100 W lamp, a Neofluar 100 x objective 1.25 x additional magnification and filter sets for blue (BP450–490, FT510, LP520) and green light excitation (LP510–560, FT580, LP590).

### RNA extraction and transcription analysis

The RNA extraction was carried out from the broth cultures of the strain of *V.limneticus* sp. nov. cultivated in the presence and absence of nitrogen. Aliquots of the all replicates were analyzed for the abundance of *V.limneticus* sp. nov. by flow cytometry as described above. A volume of 20 ml of culture correspondent to 10^8^ cells ml^− 1^ was centrifuged for 5 min at 13000 g and the pellets were processed for the RNA extraction using a commercial kit following the manufacturer’s instructions (RNeasy Protect Bacteria Mini Kit, Qiagen) with some modifications. The chemical and mechanical lysis were conducted together (4 cycles of 6000 rpm for 30s, using the Precellys 24 homogenizer, Bertin technology). Moreover, a DNase (RNase-Free DNase, Qiagen) treatment was carried out before the RNA purification. The RNA concentration was evaluated by fluorometric approach using Qubit assay following the manufacturer’s instructions (Qubit RNA HS Assay Kit, Invitrogen Life Technology). Comparable amounts of RNA (around 50 ng) of each sample were retro-transcribed following the manufacturer’s protocol (QuantiTect Reverse Transcription, Qiagen). NORT for each sample was used as non-template control for the estimation of genomic DNA amplification signal. All the samples of cDNA and NORT were tested for the presence/abundance of the nitrogenase genes (*nifHDK*) and of 16S rDNA of *V.limneticus* sp. nov. by real time PCR. The primers for all the tested genes were designed using the genome sequence of *V.limneticus* sp. nov. as template using NetPrimer software (http://www.premierbiosoft.com/netprimer/index.html), the specificity of each primer was verified by blasting all the primer sequences against that of the *V.limneticus* sp. nov. full genome. The primer sequences are reported in supplementary material (Additional file 7: Table S4). All real time PCR assays were carried out using RT-thermocycler CFX Connect (Bio-Rad), following the same programs described elsewhere [67], except for changing the annealing temperatures (Additional file 7: Table S4). In all the real time PCR assays DNA extracted from *V.limneticus* sp. nov. was used as positive control. The specificity of the reaction for each sample was ensured by the melting profile analysis using the PRECISION MELT ANALYSIS Software 1.2 built in CFX MANAGER Software 3.1 (Biorad) and by the electrophoresis gel run. The abundance of each gene for all the samples was expressed as CT value.

## Additional files

Additional file 1: **Table S1.** Main characteristics of Lake Albano (Central Italy) from where *V.limneticus* sp. nov. was isolated. A more detailed study on water chemistry is published in Ellwood et al. 2009. This volcanic lake has trace level oxygen concentration in deep water (below 30 m), increase of NH_4_-N concentration below 70 m and calcite precipitation events in spring. (PDF 182 kb)

Additional file 2: **Fig. S2.** Growth experiment of *V.limneticus* sp. nov. kept in two culture conditions: with and without nitrogen in the culture medium. The dark periods are reported as grey area. The number of cells are reported as mean of 3 replicates ± standard deviation. (PDF 337 kb)

Additional file 3: **Table S2.** ANI (Average Nucleotide Identity, %), AAI (Average Amino acid Identity, %) and GGDH (Genome-to-Genome DNA Hybridization, %, expressed as identities/HSP length) between *Vulcanococcus limneticus* sp. nov. and phylogenetically closest species. (PDF 88 kb)

Additional file 4: **Fig. S1.** Metagenomic fragment recruitment of *V.limneticus* sp. nov. concatenated genome on Amazon lake metagenomic datasets. A) Recruitment plot on Lake Ananá. B) Recruitment plot on Mancapuru Great lake. LPS biosynthesis and nif operon genomic islands are highlighted in red. (PDF 1778 kb)

Additional file 5: **Table S3.** Abundance of the genes tested by real time PCR expressed as threshold cycle value. (PDF 83 kb)

Additional file 6: **Fig. S3.** Microphotography at epifluorescence microscopy of *V.limneticus* sp. nov. cultures in the two treatments with (+N) and whithout (-N) nitrogen. Glycogen granules are clearly visible in the N limited culture. (Zeiss Axioplan, 1250×, blue excitation). *V.limneticus* sp. nov. monoclonal culture was isolated from a volcanic freshwater mesotrophic lake in central Italy, Lake Albano. Dimension of cells: 0.97 ± 0.21 × 0.76 ± 0.12 μm, Volume: 0.36 ± 0.19 μm^3^. (PDF 644 kb)

Additional file 7: **Table S4.** Primer pairs used to quantify 16S rDNA and nif genes of *V.limneticus* sp. nov. (PDF 163 kb)

## Abbreviations

AAI: Average Amino acid Identity
ANI: Average Nucleotide Identity
CT: Threshold Cycle
CynS: Cyanate hydratase
*fdx*: Ferredoxin
FSC: Forward light scatter
GGDH: Genome-to-Genome DNA Hybridization
HGT: Horizontal Gene Transfer
HoxFUYH: Bidirectional hydrogenase
HUP: Hydrogenase Uptake activity
HupSL: Uptake hydrogenase
*modAB*: molybdenum transporter
NAG: N-acetylglucosamine utilization
*nif*: Nitrogen fixing
NORT: NOn-Reverse Transcribed RNA
PE: Phycoerythrin
SSC: Side light scatter
ssuEADCB: Monooxygenases sulfonate ABC transporter
TauABCD: Alakanesulfonate ABC transporter
XRE: Xenobiotic Response Element
