# Supplementary material for: Genome analysis of the freshwater planktonic Vulcanococcus limneticus sp. nov. reveals horizontal transfer of nitrogenase operon and alternative pathways of nitrogen utilization
Source: BMC Genomics. 2018 Apr 16;19:259. doi: 10.1186/s12864-018-4648-3 (PMC5902973; doi:10.1186/s12864-018-4648-3)
Supplement: Supplementary file 1 — Table S1. Main characteristics of Lake Albano (Central Italy) from where V.limneticus sp. nov. was isolated. A more detailed study on water chemistry is published in Ellwood et al. 2009. This volcanic lake has trace level oxygen concentration in deep water (below 30 m), increase of NH4-N concentration below 70 m and calcite precipitation events in spring. (PDF 182 kb) [file 12864_2018_4648_MOESM1_ESM.pdf]

Table S1. Main characteristics of Lake Albano (Central Italy) from where *Vulcanococcus limneticus* sp. nov. was isolated. A more detailed study on water chemistry is published in Ellwood *et al.* 2009. This volcanic lake has trace level oxygen concentration in deep water (below 30 m), increase of NH_4-N concentration below 70 m and calcite precipitation events in spring. N.T.W. Ellwood, P. Albertano, R. Galvez, R. Mosello, J. Limnol., 68(2):288-303, 2009.

Strain	*V. limneticus* sp. nov.
Lake of origin	Albano
Depth of origin (m)	0.5
Latitude N	41°45'
Longitude E	12°40'
Altitude (m a.s.l.)	291
Area (km^2)	6.0
Maximum depth (m)	170
pH	8.5
Epilimnetic temperature (°C)	7.5-14
Total phosphorus ($\mu g\ L^{-1}$)	40
Total nitrogen ($\mu g\ L^{-1}$)	400
Lake origin	volcanic
