# Supplementary material for: Genome analysis of the freshwater planktonic Vulcanococcus limneticus sp. nov. reveals horizontal transfer of nitrogenase operon and alternative pathways of nitrogen utilization
Source: BMC Genomics. 2018 Apr 16;19:259. doi: 10.1186/s12864-018-4648-3 (PMC5902973; doi:10.1186/s12864-018-4648-3)
Supplement: Supplementary file 2 — Fig. S2. Growth experiment of V.limneticus sp. nov. kept in two culture conditions: with and without nitrogen in the culture medium. The dark periods are reported as grey area. The number of cells are reported as mean of 3 replicates ± standard deviation. (PDF 337 kb) [file 12864_2018_4648_MOESM2_ESM.pdf]

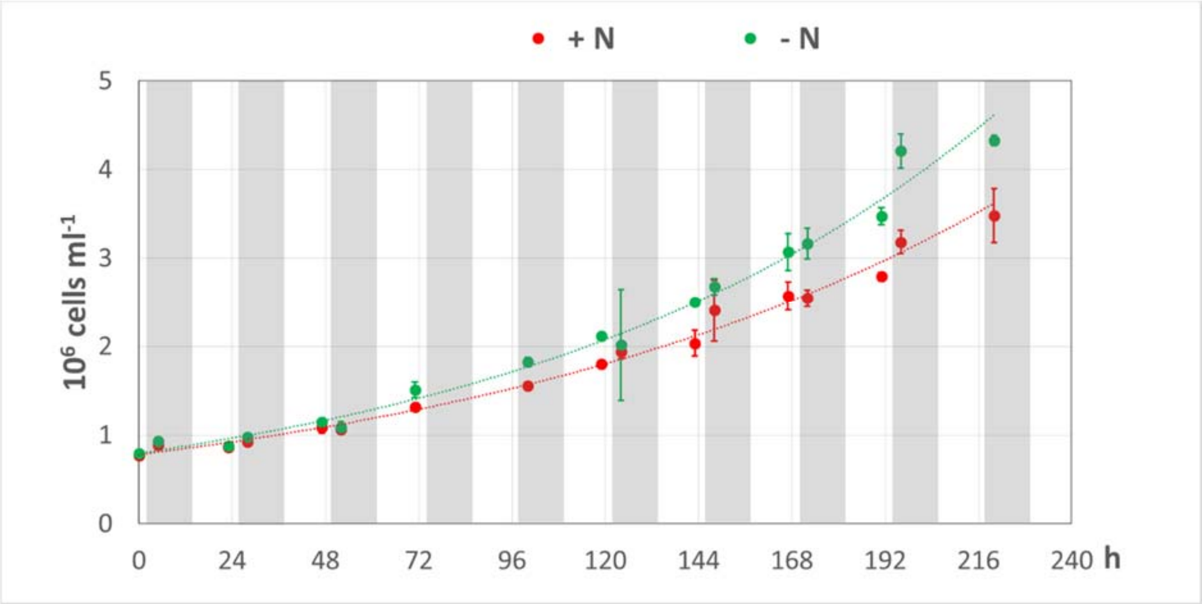

Fig. S2. Growth experiment of *Vulcanococcus limneticus* sp. nov. kept in two culture conditions: with and without nitrogen in the culture medium. The dark periods are reported as grey area. The number of cells are reported as mean of 3 replicates ± standard deviation.
