# Supplementary material for: Genome analysis of the freshwater planktonic Vulcanococcus limneticus sp. nov. reveals horizontal transfer of nitrogenase operon and alternative pathways of nitrogen utilization
Source: BMC Genomics. 2018 Apr 16;19:259. doi: 10.1186/s12864-018-4648-3 (PMC5902973; doi:10.1186/s12864-018-4648-3)
Supplement: Supplementary file 3 — Table S2. ANI (Average Nucleotide Identity, %), AAI (Average Amino acid Identity, %) and GGDH (Genome-to-Genome DNA Hybridization, %, expressed as identities/HSP length) between Vulcanococcus limneticus sp. nov. and phylogenetically closest species. (PDF 88 kb) [file 12864_2018_4648_MOESM3_ESM.pdf]

Table S2. ANI (Average Nucleotide Identity, %), AAI (Average Amino acid Identity, %) and GGDH (Genome-to-Genome DNA Hybridization, %, expressed as identities/HSP length) between *Vulcanococcus limneticus* sp. nov. and phylogenetically closest species.

Strain	ANI (%)	AAI (%)	GGDH (%)
Synechococcus sp. CB0101/*Magnicoccus sudiatlanticus* CB0101	77.65	68.42	22.1
Synechococcus sp. CB0205/ *Magnicoccus indicus* CB0205	76	67.44	21.4
Cyanobacterium sp. CACIAM 14	76.11	69.59	20.7
Cyanobium gracile PCC 6307	76.91	70.07	21.3
Cyanobium PCC 7001	77.14	68.3	21.5
Synechococcus sp. 1G10	75.6	70.37	20.4
Synechococcus sp. 8F6	77.74	70.15	22.4
Synechococcus sp. BO8801	77.14	69.78	21.3
Synechococcus sp. GFB01	78.15	71.34	22.8
Synechococcus sp. MW101C3	76.16	70.99	20.7
Synechococcus sp. SynAce01	75.03	68.12	20.3
Synechococcus sp. WH 5701/*Regnicoccus antarticus* WH5701	76.28	70.61	20.7
