# Supplementary material for: Genome analysis of the freshwater planktonic Vulcanococcus limneticus sp. nov. reveals horizontal transfer of nitrogenase operon and alternative pathways of nitrogen utilization
Source: BMC Genomics. 2018 Apr 16;19:259. doi: 10.1186/s12864-018-4648-3 (PMC5902973; doi:10.1186/s12864-018-4648-3)
Supplement: Supplementary file 4 — Fig. S1. Metagenomic fragment recruitment of V.limneticus sp. nov. concatenated genome on Amazon lake metagenomic datasets. A) Recruitment plot on Lake Ananá. B) Recruitment plot on Mancapuru Great lake. LPS biosynthesis and nif operon genomic islands are highlighted in red. (PDF 1778 kb) [file 12864_2018_4648_MOESM4_ESM.pdf]

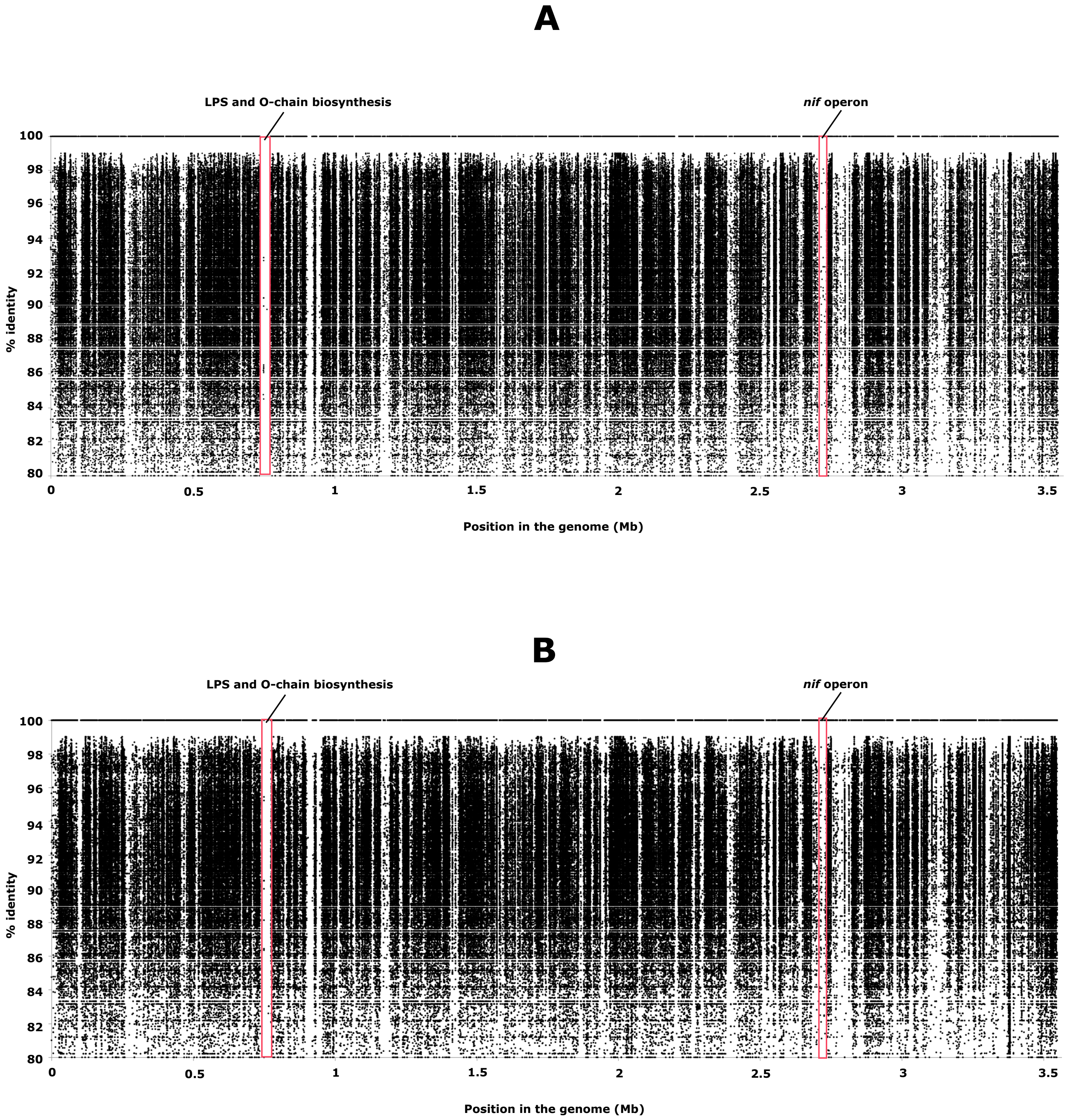


Fig S1. Metagenomic fragment recruitment of *Vulcanococcus limneticus* sp. nov. concatenated genome on Amazon Lakes metagenomic datasets. A) Recruitment plot on Lake Ananá. B) Recruitment plot on Mancapuru Great Lake. LPS biosynthesis and *nif* operon genomic islands are highlighted in red.
