# Supplementary material for: Genome analysis of the freshwater planktonic Vulcanococcus limneticus sp. nov. reveals horizontal transfer of nitrogenase operon and alternative pathways of nitrogen utilization
Source: BMC Genomics. 2018 Apr 16;19:259. doi: 10.1186/s12864-018-4648-3 (PMC5902973; doi:10.1186/s12864-018-4648-3)
Supplement: Supplementary file 5 — Table S3. Abundance of the genes tested by real time PCR expressed as threshold cycle value. (PDF 83 kb) [file 12864_2018_4648_MOESM5_ESM.pdf]

Table S3. Abundance of the genes tested by real time PCR expressed as threshold cycle value.

Presence of Nitrogen	Replicate	16SrDNA	*nifK*	*nifD*	*nifH*
-	1	19.4	34.8	31.4	33.4
-	1NORT	negative	34.1	31.4	33.3
-	2	18.9	34.5	33.2	negative
-	2NORT	negative	negative	34.7	32.2
-	3	18.7	32.2	31.9	negative
-	3NORT	32.7	negative	32.0	33.4
+	1	19.2	34.4	31.9	33.9
+	1NORT	34.2	34.3	32.0	33.0
+	2	19.2	34.4	32.6	negative
+	2NORT	31.7	negative	negative	negative
+	3	19.2	negative	34.7	negative
+	3NORT	34.8	34.8	33.3	33.5
