# Supplementary material for: Genome analysis of the freshwater planktonic Vulcanococcus limneticus sp. nov. reveals horizontal transfer of nitrogenase operon and alternative pathways of nitrogen utilization
Source: BMC Genomics. 2018 Apr 16;19:259. doi: 10.1186/s12864-018-4648-3 (PMC5902973; doi:10.1186/s12864-018-4648-3)
Supplement: Supplementary file 6 — Fig. S3. Microphotography at epifluorescence microscopy of V.limneticus sp. nov. cultures in the two treatments with (+N) and whithout (-N) nitrogen. Glycogen granules are clearly visible in the N limited culture. (Zeiss Axioplan, 1250×, blue excitation). V.limneticus sp. nov. monoclonal culture was isolated from a volcanic freshwater mesotrophic lake in central Italy, Lake Albano. Dimension of cells: 0.97 ± 0.21 × 0.76 ± 0.12 μm, Volume: 0.36 ± 0.19 μm3. (PDF 644 kb) [file 12864_2018_4648_MOESM6_ESM.pdf]

+N *Vulcanococcus limneticus* sp. nov. **-N *Vulcanococcus limneticus* sp. nov.**

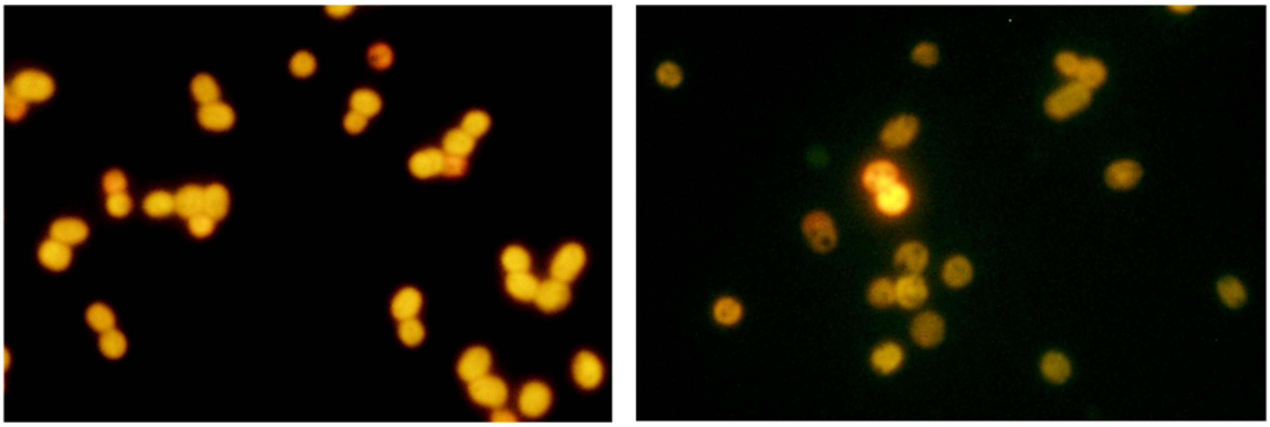

Fig. S3. Microphotography at epifluorescence microscopy of *Vulcanococcus limneticus* sp. nov. cultures in the two treatments with (+N) and whithout (-N) nitrogen. Glycogen granules are clearly visible in the N limited culture. (Zeiss Axioplan, 1250x, blue excitation). *Vulcanococcus limneticus* sp. nov. monoclonal culture was isolated from a volcanic freshwater mesotrophic lake in central Italy, Lake Albano. Dimension of cells: 0.97±0.21x 0.76±0.12 μm, Volume: 0.36±0.19 μm^3.
