# Supplementary material for: Genome analysis of the freshwater planktonic Vulcanococcus limneticus sp. nov. reveals horizontal transfer of nitrogenase operon and alternative pathways of nitrogen utilization
Source: BMC Genomics. 2018 Apr 16;19:259. doi: 10.1186/s12864-018-4648-3 (PMC5902973; doi:10.1186/s12864-018-4648-3)
Supplement: Supplementary file 7 — Table S4. Primer pairs used to quantify 16S rDNA and nif genes of V.limneticus sp. nov. (PDF 163 kb) [file 12864_2018_4648_MOESM7_ESM.pdf]

Table S4. Primer pairs used to quantify 16S rDNA and *nif* genes of *Vulcanococcus limneticus* sp. nov.

Target Name	Primer sequence (5’- 3’)	Amplicon size (bp)	Annealing temperature (°C)
16S rDNA *V. limneticus* sp. nov.	CGTGGCTCAGTTCAGATCGTA<br>TTCGGCATCCTCCTCCCTTAC	181	60
nifH	GGAAGAAAATGGTGCCTAC<br>CGAATTGGCATGGCGAAGC	90	58
nifD	GTTCACTTCCGATTTCCAGG<br>CCGCTTCAATATCATCACCG	159	58
nifK	ATCTGGGGCGGCAAGGACC<br>GCCATAGGTGCTGTAGCGG	177	60
